# No Evidence of Systematic Change of Physical Activity Patterns Before and During the Covid-19 Pandemic and Related Mood States Among Iranian Adults Attending Team Sports Activities

**DOI:** 10.3389/fpsyg.2021.641895

**Published:** 2021-04-14

**Authors:** Alireza Aghababa, Seyed Hojjat Zamani Sani, Hadi Rohani, Maghsoud Nabilpour, Georgian Badicu, Zahra Fathirezaie, Serge Brand

**Affiliations:** ^1^Department of Sport Psychology, Sport Sciences Research Institute, Tehran, Iran; ^2^Department of Motor Behavior, Physical Education and Sport Sciences Faculty, University of Tabriz, Tabriz, Iran; ^3^Department of Exercise Physiology, Sport Sciences Research Institute, Tehran, Iran; ^4^Department of Sports Physiology, Faculty of Educational Sciences and Psychology, Mohaghegh Ardabili University, Ardabil, Iran; ^5^Department of Physical Education and Special Motricity, Faculty of Physical Education and Mountain Sports, Transilvania University of Brasov, Brasov, Romania; ^6^Motor Behavior Faculty, Physical Education and Sport Sciences Faculty, University of Tabriz, Tabriz, Iran; ^7^Division of Sport Science, Department of Sport, Exercise and Health, University of Basel, Basel, Switzerland; ^8^Adult Psychiatric Clinics (UPKE), Center for Affective, Stress and Sleep Disorders (ZASS), University of Basel, Basel, Switzerland; ^9^Substance Use Prevention Research Center and Sleep Disorder Research Center, Kermanshah University of Medical Sciences, Kermanshah, Iran; ^10^School of Medicine, Tehran University of Medical Sciences, Tehran, Iran

**Keywords:** COVID-19 pandemic, team sports, sex differences, physical activity, mood states

## Abstract

**Objective:** To cope with the Coronavirus Disease 2019 (COVID-19) pandemic health authorities released social restrictions. Such social restrictions impacted on the people's possibilities to move deliberately in a public space and to gather with other people. In the present study, we investigated the impact of COVID-19-related restrictions (“confinement”) on physical activity (PA) patterns before and during the confinement among team sports participants. Such PA patterns were further related to current mood states, and possible sex differences were also explored.

**Methods:** A total of 476 adults exercising team sport (football, futsal, volleyball, handball, and basketball; mean age: 24.66 years; 48.1% females) completed a series of self-rating questionnaires covering sociodemographic information, former and current PA patterns, and current mood states.

**Results:** Compared with the period before the confinement, PA intensity decreased, but PA frequency increased during the confinement. Past, current, and changes in physical activity patterns were unrelated to participants' mood states. Sex differences in mood were spurious. Sex differences in physical activity patterns were modest, with male participants reporting a higher physical activity intensity during the confinement.

**Conclusions:** The present pattern of results suggests that the COVID-19-related confinement did not impact in a uniform fashion on PA patterns of adults attending team sports. Furthermore, mood states were unrelated to current physical activity patterns. Given the complex psychosocial situation of COVID-19-related confinement, it appeared very unlikely that sole physical activity patterns could counterbalance possible impaired states of mood and behavior.

## Introduction

By the end of June 2020, the emergence of the Coronavirus Disease 2019 (COVID-19) caused over 8.5 million cases and 456,973 deaths worldwide. Likewise, 200,262 cases and 9,392 deaths have been recorded in Iran by the end of June (World Health Organization, 2020). By the end of October 2020, Iran reported up to 490 COVID-19-related deaths per day (COVID-19 Resources, 2020). The virus with its epidemic danger forced health care authorities to act. Specifically, to decrease the risk of spreading further the virus and to cause further deaths and severely infected people, health authorities imposed confinements. To this end, health authorities legislated temporarily to close borders, schools, universities, cultural and sports events, and to disallow gatherings in open spaces. Moving in an open space (e.g., going to work, health care, grocery shopping, and exercising) was associated with rigorously following rules such as social distancing, avoiding direct contact with other people and objects, wearing masks and disposable gloves, and regular hand disinfection to avoid infection, quarantine in case of doubts to be infected, and isolation in case of approved infection.

Given this package of measures, it appeared plausible that people's movement and physical activity patterns decreased (Caputo and Reichert, 2020; Mehrsafar et al., 2020). To illustrate, results of the “Effects of home Confinement on multiple Lifestyle Behaviors during the COVID-19 outbreak (ECLB-COVID19)” Survey showed that home confinement had a negative effect on PA intensity levels (Ammar et al., 2020); mean physical activity levels decreased, sitting time increased, and eating behavior got unhealthier (more frequent and higher-caloric food intake). During the confinement, eating-disordered behavior increased, and physical activity levels decreased in both the general population and in individuals with eating disorders (Phillipou et al., 2020). Lower educational levels and the lack of using online tools were associated with lower physical activity levels (Constandt et al., 2020). Longitudinally, physical activity levels and sleep quality decreased, and emotional issues increased among a smaller sample of Chinese students (Zhang et al., 2020). However, previous results on the association between physical activity patterns and confinement conditions were not uniform. Among 1,098 adults, 40.5% of modestly active people got even more inactive, 22.4% of active people got less active, whereas 33% of modestly active people got more active, and 40.3% of active people got even more active (Lesser and Nienhuis, 2020). Furthermore, inactive people reporting higher outdoor physical activity engagements also reported lower anxiety scores, compared with inactive people remaining physically inactive. Adolescents with diabetes I neither increased nor decreased their physical activity levels and reported no impairments (or improvements) in physical and psychological health status (Tornese et al., 2020). Among a very large sample of Belgian adults (*N* = 13,515) those aged younger than 55 years and being physically modestly active got physically more active during the lockdown (Constandt et al., 2020).

Overall, we noted that a COVID-19-related confinement was not necessarily associated with lower physical activity levels among the general population. Furthermore, we also noted that longitudinal data appear to be missing, and this holds particularly true for studies in Iran. From the current literature on team sport physical activity patterns during the confinement, the only study retrieved so far (Constandt et al., 2020) showed that physical activity levels decreased among those adults who used to exercise with friends in a sports club. This result suggested that team sport participants decreased their PA levels, when compared with PA levels before the confinement. Thus, the first aim of the present study was to investigate the PA levels of team sports participants before and during the confinement.

It is general and common knowledge that mental health and regular physical activity are interrelated among people of both non-clinical (Ekelund et al., 2016) and clinical samples (Stubbs et al., 2018a,b). The concept of “exercise is medicine” reflects such knowledge (Gerber et al., 2016). As such, to illustrate, among a sample of females with major depressive disorders, higher expert-paced PA intensity levels improved positive mood more intensively, compared with self-paced PA intensity levels (Meyer et al., 2016). Given this background, it was conceivable that a confinement-related change in PA levels was associated with a decrease in mood. The second aim of the present study was therefore to investigate, if and to what extent current PA levels during the COVID-19 confinement were associated with current mood states.

Psychological health appears particularly important and critical during the confinement. About 35% of adults reported psychological distress, with females showing significantly higher psychological distress than males (Wind and Komproe, 2012; Sareen et al., 2013; Lowe et al., 2015; Ammar et al., 2020; Mattioli et al., 2020; Pieh et al., 2020; Qiu et al., 2020). Given this, the third aim of the present study was to investigate sex differences of PA and mood states.

To summarize, the COVID-19 pandemic forced health authorities to impose confinement regulations to reduce the risk of spreading the virus. Social distancing and closing public and educational areas such as gyms and sports facilities were introduced. No research has focused if and to what extent past and current physical activity patterns were associated with current mood states among Iranian adults attending team sports. Furthermore, possible sex differences were not explored so far. The present study was performed to counterbalance this lack of such research and to get further insight into the relation between physical activity indices and mood states before and during the confinement among Iranian adults.

The following exploratory research question and two hypotheses were formulated. First, we explored if and to what extend current physical activity patterns under the context of confinement changed compared with the period prior to the confinement: This exploratory question is justified as follows: While some findings showed a decrease in physical activity levels (Ammar et al., 2020; Caputo and Reichert, 2020; Zhang et al., 2020), others did not (Constandt et al., 2020; Lesser and Nienhuis, 2020; Tornese et al., 2020). Second, following the concept of a healthy mind in a healthy body, we assumed that changes in physical activity patterns would be associated with changes in mood states. Third, based on previous studies (Sareen et al., 2013; Pieh et al., 2020; Qiu et al., 2020), we assumed that compared with male participants, female participants would report lower physical activity levels. We hold that the present findings might be of practical and clinical importance to assist adults more thoroughly during the confinement.

## Methods

### Procedure

Adults were approached via social network sites (SNS) of team sports sites (football, futsal, volleyball, handball, and basketball) to participate at the present online survey on past and current PA patterns and mood states. On the first page of the online survey, participants were informed about the aims of the study, the anonymous data gathering, the confidential data handling, and the ethical approval of the study. Next, to provide the informed consent, participants clicked the box of agreement. The Human Research Ethics Board at the Sport Sciences Research Institute of Iran (approval ID: IR.SSRC.REC.1399.070) approved the study, which was performed in accordance to the seventh and current revision (World Medical Association, 2013) of the Declaration of Helsinki.

### Participants

While 508 participants began the online survey from March 5 to April 2, 2020, 32 (6.30%) responses were incomplete and therefore were removed from the dataset. The final sample consisted of 476 Iranian adults [48.2% males; mean age: 27.32 years (SD = 10.06 years, range = 18–54 years); 51.8% females; mean age: 22.36 years (SD = 10.18 years, range = 20–84 years)]. They were amateurs or semi-professional athletes from provincial leagues. Participants reported the following team sports: Football (*n* = 100), futsal (*n* = 100), volleyball (*n* = 100), handball (*n* = 76), and basketball (*n* = 100).

## Measures

### Sociodemographic Information

Participants reported on their age (years), sex (female, male), civil status (single, divorced, widowed), highest educational level (diploma, high school, and higher education), and current job situation (working: employed or self-employed; not working: unemployed; retired; student).

### Physical Activity Levels

Participants answered the following questions: 1. “Which team sport did you attend before the confinement?” 2. “How many days per week did you attend the team sport?” 3. “How many days per week do you currently attend the team sport?” The answers were everyday; 6 days/week; 5 days/week; 4 days/week; 3 days/week; 2 days/week; 1 day/week; and currently off. 4. “Indicate the intensity of exercising before the confinement” and “Indicate the current intensity of exercising.” The answers were low intensity, moderate intensity, vigorous intensity, and very vigorous intensity. All items are taken from another study (Cho, 2014). Cho reported satisfactory psychometric properties of the questionnaire.

### Mood State

To assess both positive and negative mood states, participants completed the shortened version of the Brunel Mood Scale (BRUMS) (Terry et al., 2003). The following mood states were asked: positive mood states: lively, alertness, active, vigorous; negative mood states: angry, worn out, uncertain about things, grouchy, hopeless, fatigued, annoyed, discouraged, exhausted, gloomy, weary, and furious. Answers are given on five-point Likert scales ranging from 0 (=no) to 4 (=extremely); higher scores reflect a more pronounced mood state (Cronbach's alpha: 0.90).

### Statistical Methods

With a series of *X*^2^- and Cramér's *V*-tests, sociodemographic information was compared between female and male participants. A series of Pearson's correlations was performed associate physical activity pattern before and during the confinement with positive and negative mood states. The level of significance was set at alpha < 0.05. All statistical analyses were computed utilizing SPSS® 23.0 (IBM Corporation, Armonk NY, USA) for Windows®.

## Results

### Sociodemographic Information and Sex

Table 1 provides the descriptive and inferential statistical indices of sociodemographic information between female and male participants. Compared with the female participants, the male participants were older, they were more often married, and reported higher education levels; furthermore, male participants were more often retired/unable to work or unemployed.

**Table 1:** Participants' demographic characteristics, separately for male and female participants.

	**Total**	**Female**	**Male**	**Statistics**
*N*	476	229	247	
Age (mean ± SD)	24.66 ± 10.28	22.36 ± 10.18	27.32 ± 10.06	*t*_(475)_ = 5.25^***^
Marital status *n* (%)				*X*^2^ (*N* = 313; df = 3) = 12.077^**^; Cramer's *V* = 0.16
Single	224 (47.06)	124 (26.05)	100 (21.01)	
Married	84 (17.65)	28 (5.89)	56 (11.76)	
Separated	5 (1.05)	3 (0.63)	2 (0.42)	
Missing data = 163 (34.24)				
Education level *n* (%)				*X*^2^ (*N* = 459, df = 3) = 32.04^***^; Cramer's *V* = 0.9
Higher education	165 (34.67)	58 (12.19)	107 (22.48)	
Diploma	138 (28.99)	81 (17.02)	57 (11.97)	
High school or lower	156 (32.77)	95 (19.96)	61 (12.81)	
Missing data = 17 (3.57)				
Employment status *n* (%)				*X*^2^ (*N* = 458; df = 3) = 25.26^***^ Cramer's *V* = 0.21
Retired or unable to work	9 (1.90)	3 (0.63)	6 (1.27)	
Unemployed	33 (6.93)	12 (2.52)	21 (4.41)	
Employed	416 (87.39)	219 (46)	197 (41.39)	
Missing data = 18 (3.78)				
Family income *n* (%)				*X*^2^ (*N* = 412; df = 3) = 128
High	42 (8.82)	19 (3.99)	23 (4.83)	
Moderate	176 (36.98)	84 (17.65)	92 (19.33)	
Low	194 (40.76)	98 (20.59)	96 (20.17)	
Missing data or I don't know = 64 (13.44)				

** p < 0.01;

*** *p < 0.001*.

### Physical Activity (Intensity; Frequency) Before and During the Confinement

Tables 2, 3 provide the descriptive and inferential statistical overview of physical activity patterns (intensity, frequency) before and during the confinement, and between female and male participants.

**Table 2:** Intensity of physical activity (PA) before and during the coronavirus disease 2019 (COVID-19), and separately for female and male participants.

**Intensity of PA**	**Before COVID-19**			**During COVID-19**		
	**Female *n* (%)**	**Male *n* (%)**	**Total**	**Female *n* (%)**	**Male *n* (%)**	**Total**
Low	14 (2.94)	17 (3.57)	31 (6.51)	65 (13.65)	69 (14.50)	134 (28.15)
Moderate	76 (15.97)	72 (15.13)	148 (31.1)	138 (28.99)	97 (20.37)	235 (49.36)
High	113 (23.74)	104 (21.85)	217 (45.59)	18 (3.78)	37 (7.77)	55 (11.55)
Very high	27 (5.67)	31 (6.51)	58 (12.18)	2 (0.42)	11 (2.31)	13 (2.73)
	*X*^2^ (*N* = 454; df = 4) = 0.968			*X*^2^(*N* = 437, df = 4) = 19.89^***^		

*** *p < 0.001*.

**Table 3:** Frequency of PA before and during COVID-19, and separately for female and male participants.

	**Before COVID-19**			**During COVID-19**		
**Frequency of PA**	**Female *n* (%)**	**Male *n* (%)**	**Total *n* (%)**	**Female *n* (%)**	**Male *n* (%)**	**Total *n* (%)**
Never	0	0	0	22 (4.62)	25 (5.26)	47 (9.88)
1 day per week	12 (2.52)	85 (17.86)	97 (20.38)	23 (4.84)	21 (4.41)	44 (9.25)
2 days per week	20 (4.20)	75 (15.76)	95 (19.96)	30 (6.30)	21 (4.41)	51 (10.71)
3 days per week	71 (14.91)	28 (5.88)	99 (20.79)	36 (7.56)	27 (5.67)	63 (13.23)
4 days per week	59 (12.40)	13 (2.73)	72 (15.13)	8 (1.68)	13 (2.73)	21 (4.59)
5 days per week	72 (15.13)	24 (5.04)	96 (20.17)	8 (1.68)	10 (2.11)	18 (3.97)
6days per week	0	0	0	8 (1.68)	9 (1.89)	17 (3.57)
Every day	0	0	0	26 (5.46)	34 (7.14)	60 (12.60)
Statistics	*X*^2^ (*N* = 459; df = 7) = 7.57			*X*^2^(*N* = 459; df = 7) = 12.058		

Over time, PA intensity decreased during the confinement, and PA frequency remained unchanged, though sex differences were observed (see below).

### Physical Activity (Intensity; Frequency) Between Female and Male Participants

Over time, physical activity intensity decreased in both female and male participants. Over time, physical activity frequency increased in male participants, while physical activity frequency remained unchanged in female participants.

**Figure 1:**
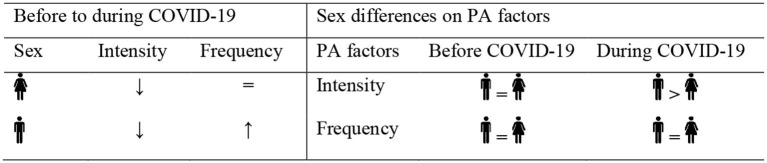
Illustration of physical activity (PA) factors and sexes interaction before and during the coronavirus disease 2019 (COVID-19).

Figure 1 summarizes the interaction between physical activity factors and sexes.

### Mood States and Sexes

Compared with female participants, male participants reported to be more grouchy, gloomy, and vigorous (all *X*^2^'s > 11.00, *p*'s < 0.05; all Cramer's *V* > 0.73). For all other mood dimensions (angry, worn out, lively, uncertain, hopeless, fatigued, annoyed, discouraged, exhausted, weary, furious, and active), no significant sex differences were observed (all *X*^2^'s < 6.40, *p*'s > 0.05).

### Relationship Between Current Mood States and Physical Activity Indices Before and During the Confinement

Correlational matrix of physical activity indices before and during the confinement and current mood states showed that physical activity indices before and during the confinement and participants' current mood states were weakly related or fully unrelated; correlation coefficients ranged between 0 and 0.19 (trivial/spurious associations), or the other way around: *r* = 0.19 = *R*^2^ = 0.036; thus, 3.6% of the variance of physical activity indices explained the variance of mood states, while 96.4% of the variance of mood states remained unexplained (Tables 4–6).

**Table 4:** Correlation coefficients between PA indices and mood states among males.

	**Before COVID-19**		**During COVID-19**	
	**Intensity**	**Frequency**	**Intensity**	**Frequency**
	***r***	***r***	***r***	***r***
Angry	0.02	−0.09	0.11	0.05
Worn out	−0.03	0.10	0.03	0.17^**^
Lively	0.03	0.08	−0.06	0.16^*^
Uncertain about things	0.01	−0.14^*^	0.02	0.10
Grouchy	−0.02	−0.09	0.06	0.09
Hopeless	0.02	−0.01	0.10	0.07
Fatigued	−0.03	−0.07	0.06	−0.01
Annoyed	0.09	−0.11	0.09	0.11
Discouraged	−0.01	−0.08	0.01	0.09
Exhausted	0.05	0.06	0.07	0.13^*^
Gloomy	0.04	−0.04	0.06	0.09
Weary	0.02	−0.14^*^	0.11	0.03
Alert	0.01	0.08	−0.09	−0.03
Furious	−0.01	−0.08	0.04	0.11
Active	0.09	0.01	0.07	0.05
Vigorous	0.04	0.01	0.03	0.03
Mood (Total)	0.03	−0.06	0.04	0.10
Mood (Positive)	0.06	0.07	−0.03	0.05
Mood (Negative)	−0.01	−0.08	0.04	0.09

* p ≤ 0.05,

** *p ≤ 0.01*.

**Table 5:** Correlation coefficients between PA indices and mood states among females.

	**Before COVID-19**		**During COVID-19**	
	**Intensity**	**Frequency**	**Intensity**	**Frequency**
	***r***	***r***	***r***	***r***
Angry	−0.11	−0.01	−0.03	0.01
Worn out	−0.11	−0.03	0.06	0.04
Lively	0.15^*^	0.08	0.11	−0.01
Uncertain about things	−0.01	−0.05	0.12	−0.03
Grouchy	−0.08	−0.01	−0.03	0.02
Hopeless	−0.07	−0.09	−0.07	−0.01
Fatigued	−0.1	−0.12	−0.01	−0.04
Annoyed	−0.08	−0.13^*^	−0.09	−0.05
Discouraged	−0.15^*^	0.09	−0.06	0.09
Exhausted	−0.02	−0.16^**^	−0.01	0.01
Gloomy	0.07	−0.06	−0.06	0.03
Weary	−0.03	−0.09	−0.11	−0.06
Alert	0.07	0.06	0.07	0.10
Furious	−0.14^*^	−0.08	−0.05	0.05
Active	0.19^**^	0.06	0.15^*^	0.09
Vigorous	0.17^**^	0.06	0.10	0.10
Mood (Total)	0.06	−0.06	−0.06	0.02
Mood (Positive)	0.18^**^	0.09	0.10	0.10
Mood (Negative)	−0.11	−0.09	−0.08	0.01

* p ≤ 0.05,

** *p ≤ 0.01*.

**Table 6:** Correlation coefficients between PA indices and mood states (total).

	**Before COVID-19**		**During COVID-19**	
	**Intensity**	**Frequency**	**Intensity**	**Frequency**
	***r***	***r***	***r***	***r***
Angry	−0.04	−0.07	0.06	0.03
Worn out	0.07	−0.01	0.01	0.06
Lively	0.11^*^	0.07	0.04	0.07
Uncertain about things	−0.02	−0.08	0.03	0.02
Grouchy	−0.05	0.11^*^	0.02	0.06
Hopeless	−0.02	0.07	0.02	0.04
Fatigued	−0.06	−0.09	0.04	−0.03
Annoyed	−0.05	−0.12	0.01	0.03
Discouraged	−0.08	−0.08	−0.02	0.09^*^
Exhausted	0.01	−0.09	0.03	0.06
Gloomy	−0.02	−0.08	0.01	0.06
Weary	−0.01	0.15^*^	0.01	−0.01
Alert	0.03	−0.02	−0.02	0.02
Furious	−0.07	0.07	0.01	0.09^*^
Active	0.14^**^	0.01	0.11^*^	0.06
Vigorous	0.12	−0.05	0.07	0.05
Mood (Total)	−0.01	−0.09	0.01	0.06
Mood (Positive)	0.13^**^	0.02	0.05	0.07
Mood (Negative)	−0.05	−0.11^*^	−0.01	0.04

* p ≤ 0.05,

** *p ≤ 0.01*.

## Discussion

The key findings of the present study were that among a larger sample of Iranian adults attending team sports activities before and during the COVID-19-related confinement physical activity intensity decreased, but physical activity frequency increased in the male participants and remained stable among the female participants. Furthermore, physical activity indices before and during the confinement were unrelated to current mood states. The present pattern of the results expands upon previous research in that we showed that a COVID-19-related confinement had a very modest impact on participants' physical activity indices and that such physical activity indices were unrelated to participants' mood states. We hold that these results are of practical importance: Against mainstream and mostly media-driven assumptions, confinement had a spurious to modest impact on adults' physical activity patterns and mood states among Iranian adults attending team sports activities.

This is the research question asked, if physical activity indices changed during the confinement, and data did not fully support this assumption: While physical activity intensity decreased, physical activity frequency increased among male participants, and remained unchanged among female participants. Accordingly, the present data are not in accord with those data reported elsewhere (Ammar et al., 2020; Caputo and Reichert, 2020; Zhang et al., 2020); rather, the present data match with the results of those studies, which were unable to find meaningful changes in physical activity indices due to the confinement (Constandt et al., 2020; Lesser and Nienhuis, 2020; Tornese et al., 2020). Given this, we hold that the present study has the potential to counteract the general and often media-driven assumption of a dramatic negative impact of the confinement on adults' life. Our results do match those observed among 1,098 Canadian adults (Lesser and Nienhuis, 2020): While 40.5% of modestly active people got even more inactive during the confinement, 33% of inactive people reported that they got much more active. Similarly, while 22.4% of the active adults got less active during the confinement, the opposite was true for 40.3% of the active people. Among the 13,515 adults under 55 years, inactive people got more active during the confinement (Constandt et al., 2020). In contrast, participants who were used to exercise with friends in a sport club and not using online tools reported decreased physical activity indices. Here, we note that the present results are even more impressive, when we consider that exclusively adults attending team sports were included in the study: By definition, attending team sports requires physical activities with other people, which is against the confinement rules of social distancing, closed sports institutions, and staying at home. The quality of the data does not allow a deeper understanding of the underlying mechanisms of respecting social distancing and current confinement rules. Given this, though highly speculatively, it is conceivable that at the time lapse of data gathering, confinement rules were adapted to that current context of danger. Similarly, it is also conceivable that people wanted to counterbalance possible unpleasant effects of confinement on general health such as weight gain, increased eating behavior (Phillipou et al., 2020), and impaired mood.

With the first hypothesis, we assumed that changes in physical activity patterns would be associated with changes in mood states. This hypothesis is based on the extant literature, which showed both correlative and causal associations between higher physical activity indices and a more favorable psychological functioning (Stubbs et al., 2018b; Vancampfort et al., 2018; Schuch et al., 2019). However, the present data did not confirm this assumption: Correlation coefficients between physical activity indices and mood states were trivial and spurious (Table 4 for males, Table 5 for females, and Table 6 for the total sample). To illustrate, about 3.6% of the variance of the physical activity indices explained the variance of mood states, while, complementarily, about 95.6% of the variance of mood states remained unexplained. Furthermore, while some correlation coefficients were statistically significant (*p* < 0.05; see Tables 4–6), such *p*-values were due to the large sample size, but not to a “true” meaningful association. Given this, physical activity indices and mood states were unrelated. While again the quality of the data does not allow a deeper understanding of the underlying social and psychological mechanisms, the present data are in line with those of a previous study: Opdal et al. (2019) were unable to find meaningful associations between changes in physical activity indices measured objectively with activity trackers and mental distress among a sample of 676, 16.5 years old adolescents over a time lapse of 2 years. Furthermore, as mentioned in the Introduction section, Iran reports a high prevalence rate of COVID-19-related death and infected people (COVID-19 Resources, 2020). Accordingly, it is conceivable that participants' mood states were more tightly related to health concerns of their social environment.

With the second hypothesis, we assumed that compared with male participants, female participants reported more concerns about their mood and physical activity states, and again, the pattern of results did not confirm this assumption: Differences in mood states were modest, and differences in physical activity patterns, too. Given this, the present findings were not in accord with results reported elsewhere: Qiu et al. (2020) cited that females have significantly higher psychological distress than males, and Sareen et al. (2013) stated that women are more affected by stressful situations. Our results among females were inconsistent with this finding. Thus, again, while the quality of the data does not allow a deeper understanding of underlying psychological mechanisms, we assume that several social, cognitive, and emotional factors completely unrelated to physical activity did shape a crucial psychological dimension such as mood.

It is possible that more important conditions during the COVID-19 pandemic such as living with relatives with COVID-19, precarious job conditions, and financial constraints might have impacted on participants' mental and physical health. It is conceivable that such concerns had a higher influence on participants' mood states; this claim is in contrast to those results observed before the COVID-19 pandemic, where PA patterns were associated with positive mood. Although PA was recommended as one of the first and best ways to keep mental health stable during the confinement (Meyer et al., 2020), the present data could not confirm this claim.

Despite the novelty and unexpected results of the present study, the following limitations should be considered. First, we did fully rely on self-reports, which, by definition, might be biased. Accordingly, objective physical activity assessments would have allowed to compare subjectively and objectively gathered physical activity patterns and to detect possible differences. Second, only adult participants willing and able to comply with the study requirements (access to the Internet, literacy, and team sports activities) completed the survey; given this, a biased selection of participants cannot be ruled out. We also note that participants remained physically active during the COVID-19 confinement, despite the fact that team sports activities were not possible. Accordingly, it is conceivable that participants of the present study were particularly motivated and self-efficient to keep their physical activity indices unaltered. Third, physical activity patterns before the confinement were assessed retrospectively; accordingly, it is conceivable that memory recall might have been biased. Fourth, it is conceivable that further unassessed psychological dimensions might have biased two or more dimensions in the same or opposite direction. To recall, about 95% of the variance of mood states remained unexplained. Fifth, we focused on adults of team sports; it is possible that adults with individual sports activities would have provided another picture of the results. Sixth, although data collection of the present research was performed only 2 months after the COVID-19 pandemic official report in Iran, a longitudinal design would have allowed to describe causal relationships between physical activity pattern and mood states. Finally, at the time of data collection, international sanctions imposed to Iran led to severe economic problems, which, in turn, impacted negatively on people's quality of life.

## Conclusion

The main aim of our research was to provide new insight on the PA level of adults attending team sports activities before and during the COVID-19 confinement and their related mood states. Our results suggested that there is no evidence of systematic change in physical activity patterns before and during the COVID-19 confinement and the related mood states among adults attending team sports activities: While PA intensity decreased, PA frequency increased during the COVID-19 confinement. Mood states were unrelated to physical activity patterns. Also, mixed mood states were reported among males and females.

## Data Availability Statement

The raw data supporting the conclusions of this article will be made available by the authors, without undue reservation.

## Ethics Statement

The studies involving human participants were reviewed and approved by Human Research Ethics Board at the Sport Sciences Research Institute of Iran (approval ID: IR.SSRC.REC.1399.070). The patients/participants provided their written informed consent to participate in this study.

## Author Contributions

AA conceptualized the study. MN was in charge of the methodology. SZ and ZF prepared and wrote the original draft and analyzed the data. SB also analyzed the data, reviewed, edited, and wrote the final manuscript. GB also edited the manuscript, while HR supervised the study. All authors have read and agreed to the final version of the manuscript.

## Conflict of Interest

The authors declare that the research was conducted in the absence of any commercial or financial relationships that could be construed as a potential conflict of interest.
